# Early‐Life Nutritional Disruption Reshapes Gastric Epithelial Development Through Coordinated Changes in the Dynamics and Signaling of Proliferative Niches

**DOI:** 10.1002/jcp.70234

**Published:** 2026-09-25

**Authors:** Isadora Campos Rattes, Marcos Aurélio Santos da Costa, Viviane Rosa de Oliveira, Patrícia Gama

**Affiliations:** ^1^ Department of Cell and Developmental Biology, Institute of Biomedical Sciences University of Sao Paulo São Paulo Brazil

**Keywords:** breastfeeding, cell migration, early weaning, gastric stem cell, notch, Wnt

## Abstract

Gastric gland cells originate from two proliferative niches: the main rapid one in the isthmus, and the slow secondary one in the base. However, while nutrition is dependent on breastfeeding, the proliferative niches are not established, and proliferation occurs along the gland. Currently, we investigated the influence of early weaning (EW) on shaping gastric proliferative niches and their signaling. Wistar rats were separated into suckling (S) and EW groups on postnatal day (PND) 15 for gastric mucosa collection. BrdU was administered (PND 14 and 15) and as it diluted between daughter‐cells we evaluated migration and differentiation. In parallel EdU incorporation was used to identify cells during S phase. Through these tools, we studied the different profiles of BrdU/EdU dual labeling at 18, 21 and 30 PND. At 18 PND, EW decreased BrdU+ population (*p* < 0.0001 *vs*. S group) in the upper and bottom gland areas (*p* < 0.05). The BrdU + /EdU+ cells were concentrated in the upper gland, where we recorded a larger EdU+ population after EW (*p* < 0.05). On PND 30, EW decreased BrdU+ cells in the gland (*p* < 0.05), whereas EdU+ cells were more numerous in the isthmus. At 18 PND, EW reduced *Wnt3*, *Notch1*, and *Notch2* expressions (*p* < 0.05), while increasing *Bmp2*. In contrast, at 60 PND, *Axin2*, *Notch1*, and *Notch2* transcripts were elevated after EW (*p* < 0.05). Notch1 and Notch2 distribution indicated that EW might shift Notch2 to the upper gland, potentially affecting the fate of progenitor cells in pups. *In silico* analyses showed that zymogenic cells are in a renewal‐promoting environment, while parietal cells contribute to differentiation and signaling. Therefore, EW restricted the proliferative activity to the isthmus, altering the spatiotemporal expression of genes involved in stemness and differentiation, and reshaped the distribution of Notch receptors, without affecting the distribution of its downstream effector Hes1. These spatially organized molecular landscapes are crucial for proper epithelial renewal and function, and our data suggest that early nutritional disruption reprograms these niches with long‐term consequences.

## 1 Introduction

The gastric mucosa of the corpus region consists of tubular glands composed of different epithelial cell types organized as isthmus, neck and base, which are functional compartments. Their maintenance depends on the coordinated activity of proliferative cells that support continuous tissue renewal throughout life. In the past decades, different studies consistently indicated the presence of a proliferative niche in the gland isthmus of rodents and humans (Busslinger et al. 2021; Karam and Leblond 1993; Mesquita da Silva et al. 2021; Willet and Mills 2016). However, recent evidence demonstrated a secondary niche at the gland base, where slowly dividing cells can participate in regeneration and differentiation under specific conditions (Burclaff et al. 2020; Han et al. 2019; Huebner et al. 2023). This is the scenario observed in the adult gastric gland, whereas during the first three postnatal developmental weeks cell proliferation occurs along the gland mucosa in rats and mice (Mesquita da Silva et al. 2021). So, during this period, the two proliferative niches are still maturing and can be modulated by environmental factors such as dietary patterns.

The postnatal development of gastrointestinal mucosa is characterized by intense growth in rodents, and markedly, the first month is also a period of nutritional changes as weaning sets the end of suckling and the start of solid food intake. Breast milk provides nutrients, bioactive peptides and hormones that regulate epithelial proliferation and differentiation (Osaki et al. 2010; Osaki et al. 2011). Early weaning (EW), which is the abrupt interruption of breastfeeding, is a well‐established experimental model that allows the evaluation of nutritional effects on gastrointestinal development and it has been shown to induce both immediate and long‐term changes in gastric epithelial growth (Gama et al. 2000; Mesquita da Silva et al. 2021; Osaki et al. 2011).

Over the past decades, we demonstrated that EW promotes a rapid increase of proliferative indices, alters the spatial distribution of dividing cells along the gland, and anticipates an adult‐like organization (Gama et al. 2000; Ghizoni et al. 2014; Rattes et al. 2025; Teles Silva et al. 2020) Additionally, it has been shown that this response involves activation of pathways such as EGFR‐MAPK, corticosterone, and TGF‐β, along with modifications of cell cycle control, as the reduction in the levels of p27, a known cell cycle inhibitor (Mesquita da Silva et al. 2021; Osaki and Gama 2013). Furthermore, the proliferative activity and associated molecules, as the one encoded by *Tnfrsf19‐* Troy, remain influenced by EW in the adult gastric mucosa, suggesting that premature interruption of breastfeeding triggers long‐lasting epithelial reprogramming in the stomach (Rattes et al. 2025).

Despite these advances, it remains unclear how EW affects the dynamics of gastric proliferative niches during postnatal development and whether this involves changes in signaling pathways such as Notch, BMP, and Wnt, which are essential for the specification and maintenance of gland compartments, and for homeostasis (Zhang et al. 2025).

Therefore, we hypothesized that EW could anticipate the establishment of the location of proliferative niches, promoting long‐term structural and functional alterations in the rat gastric epithelium. So, in this study we aimed to investigate the effects of early weaning on the cell proliferation and migration dynamics, and on the expression of genes related to stem/progenitor cell regulation. We also studied the distribution of Notch1, Notch2, and the transcription factor Hes1 across different stages of gastric development in order to evaluate how an external element, as breastfeeding, is involved in the organization of gastric proliferative niches and their signaling.

## 2 Material and Methods

### 2.1 Animals and Experimental Design

Specific pathogen‐free Wistar rats were obtained from the breeding facility of the Institute of Biomedical Sciences (Animal Facility Network, University of São Paulo) and transferred to the animal facility at the Department of Cell and Developmental Biology. Pregnant females were housed individually under controlled temperature (22 ± 1°C), with a 12 h light/dark cycle and *ad libitum* access to water and standard chow (Nuvilab CR‐1, Quimtia, Brazil). The day of birth was designated as postnatal day (PND) 0 and litters were reduced to 8‐9 pups on PND 3. On PND 15, males and females were randomly divided into two experimental groups: suckling (S) and early weaning (EW). On being separated from their dams, EW pups were transferred to a new box containing hydrated powdered chow and water that were also offered through disposable Pasteur pipettes twice a day, when animals were also massaged to facilitate excretion, mimicking maternal care (Osaki et al. 2010). The number of pups in each group was monitored to avoid obesity and experiments proceeded when body mass was observed to change according to the patterns previously established (Teles Silva et al. 2020). Control (S) pups remained with their mothers until PND 21.

All experimental protocols were approved by the Institutional Animal Care and Use Committee (CEUA‐ICB USP: 18/2015, 115/2017, 4532180222) and followed national (CONCEA, Brazil) and international guidelines for animal experimentation.

### 2.2 BrdU/EdU Double Labeling‐ Cell Proliferation and Migration Assay

**Figure 1:**
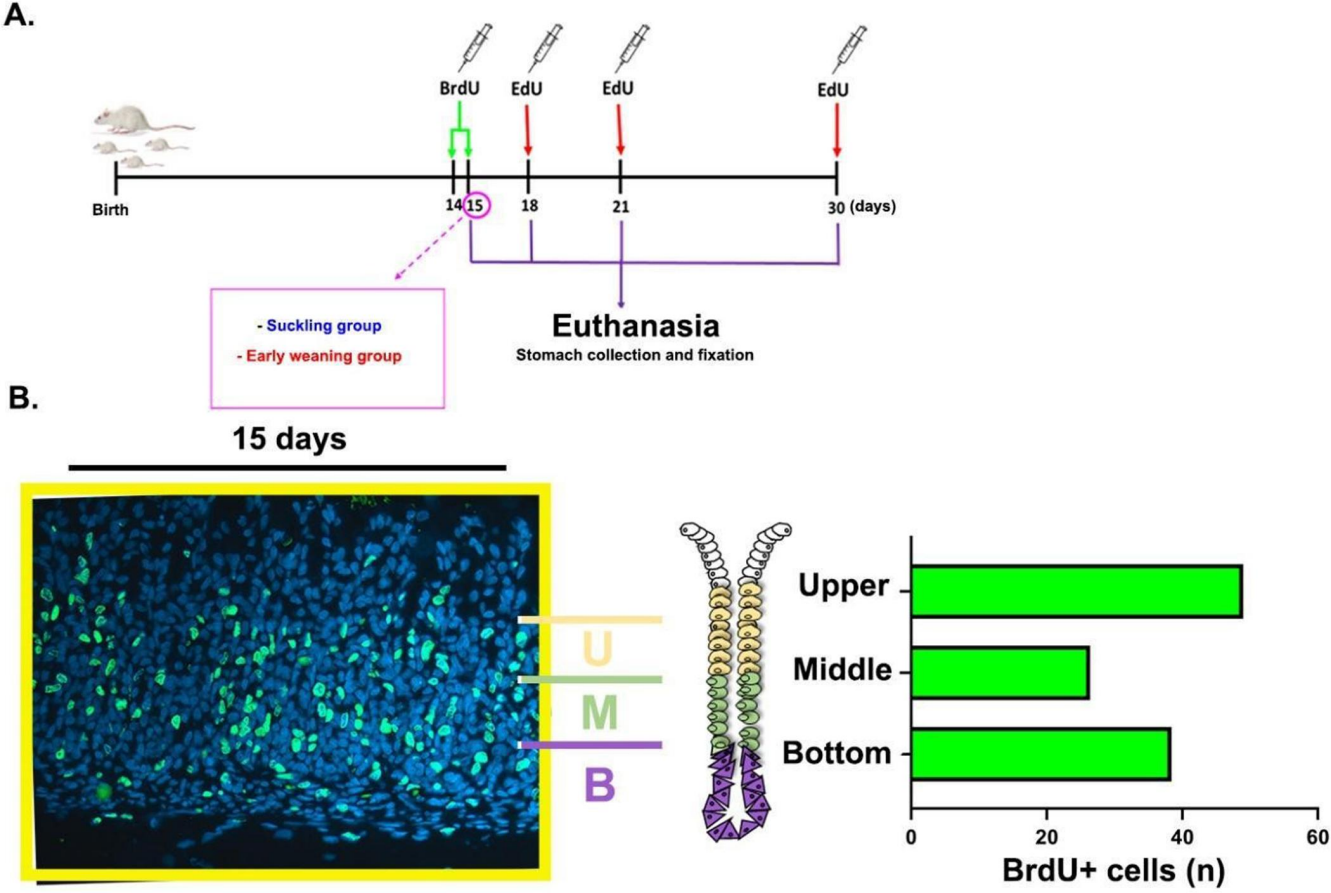
Experimental design and distribution of BrdU^+^ cells in the gastric mucosa at postnatal day 15. (A) Experimental design used from birth to postnatal day (PND) 30. Rats were assigned to the suckling (blue) or early weaning (red) groups at 15 PND. BrdU was administered on PND 14 and 15, and EdU injections were performed on PND 18, 21, and 30. At each endpoint, animals were euthanized, and the stomach was collected and fixed in 10% buffered formalin. (B) Representative image of BrdU+ cells (488 nm‐ green) in the gastric corpus mucosa at 15 PND (DAPI counterstaining, blue), when the cell kinetics study started. For quantification at 15 PND, the gastric gland was subdivided into three regions: upper (U), middle (M), and bottom (B). The bar graph on the right shows the number of BrdU^+^ cells distributed across these glandular compartments at 15 PND.

In order to track proliferative activity and cell cycle kinetics in the gland, we adapted the protocol previously described (Asano et al. 2015; Bradford and Clarke 2011), and used double labeling with 5‐bromo‐2′‐deoxyuridine (BrdU, 15 mg/mL, Sigma) and 5‐ethynyl‐2′‐deoxyuridine (EdU, 10 mM, BaseClick, Thermo Fisher Scientific) that were administered i.p. as BrdU (100 mg/kg) on PND 14 and 15, and EdU (50 mg/kg) 2 h before euthanasia, that occurred on PND 18, 21, 30 (Figure 1). The gastric corpus wall was fixed in 10% buffered formalin and embedded in paraffin. Non‐serial Section (5 μm) were processed for BrdU immunohistochemistry (BrdU Labeling and Detection Kit I ‐ Roche) and EdU detection via Click‐iT® reaction (Click‐iT™ EdU Imaging Kit with Alexa Fluor™ 564—Life Technologies), and briefly, DNA was opened with 0.1% pepsin in 0.1 N HCl (10 min, 37°C) in a water bath. After washing with PBS‐BSA, sections were incubated with Click‐iT™ cocktail with Alexa Fluor™ 594 nm (30 min in dark chamber), washed in 0.05 M PBS and incubated with anti‐BrdU (1:10, 1 h, 37°C). After development with goat secondary anti‐mice‐FITC, sections were counterstained with DAPI, (0.5 μg/mL, Invitrogen) and slides were mounted with ProLongTM Diamond Antifade Mountant (Invitrogen). Sections were observed under (Axioscope2, Zeiss; software Zen Blue). In order to detect cells that proliferated after BrdU incorporation and were in S phase in the different time points (EdU+), we identified different labeling patterns as EdU+, BrdU+ and BrdU+/EdU+. For quantification, longitudinally sectioned gastric glands were subdivided into three equal regions along the gland axis: upper (pit and isthmus), middle (neck), and base, based on relative position as previously described (Teles Silva et al. 2020; Mesquita da Silva et al. 2021; Rattes et al. 2025) in ten fields/animal in each age and condition.

### 2.3 RNA Extraction and RT‐qPCR‐ expression of Regulatory Genes

Total RNA was extracted from the scraped corpus mucosa using TRIzol® Reagent (Invitrogen) followed by purification with PureLink® RNA Mini Kit (Ambion). RNA concentration and integrity were evaluated spectrophotometrically (NanoDrop, GE). cDNA was synthesized using High‐Capacity cDNA Reverse Transcription Kit (Applied Biosystems). Quantitative real‐time PCR (RT‐qPCR, Step One Plus Thermocycler, Applied Biosystems) was performed with 40 ng of DNA using TaqMan® Gene Expression Assays (Applied Biosystems) for the following targets: *Wnt3* (Rn01506283_m1), *Axin2* (Rn01529636_g1), *Bmp2* (Rn01498206_m1), *Notch1* (Rn00566727_m1), *Notch2* (Rn01446707_m1), and *Shh* (Rn00568129_m1). Gene expression was normalized to β‐actin *(Actb*, Rn00667869_m1), and the relative expression was calculated using the 2^−ΔΔ^Ct method (Schmittgen and Livak 2008).

### 2.4 In Silico Analysis of Signaling Pathways

To assess the expression of genes related to epithelial signaling pathways during gastric development, we performed *in silico* analysis using publicly available micro‐array RNA‐sequencing data (Ramsey et al. 2007). Processed datasets were obtained from the Gene Expression Omnibus (GEO) under accession number GSE5018 (gastric epithelium microdissected fractions). Heatmaps were generated using the R2: Genomics Analysis and Visualization Platform.

### 2.5 Immunohistochemistry for Notch1 and Notch2

Formalin‐fixed, paraffin‐embedded non‐serial tissue sections at 5μm were dewaxed and rehydrated. Sections were rehydrated in 0.05 M PBS (pH 7,4) containing 0,03% Triton, and then the endogenous peroxidase activity was blocked with 3% hydrogen peroxide in methanol. Antigen retrieval was performed with 0.01 M citric acid (pH 6.0) in water‐bath in microwave (5 min at 600 W followed by 2 × 3 min at 300 W), and after slow cooling sections were washed with 0.05 M PBS and incubated with primary antibodies against Notch1 (rabbit polyclonal, 4 μg/mL, Abcam, ab27526) and Notch2 (rabbit polyclonal, 4 μg/mL, Abcam, ab8926) (overnight, 4°C). Detection was performed with HRP‐conjugated secondary antibodies (32 μg/mL Jackson ImmunoResearch Labs) followed by 0.07% 3,3'DAB (Sigma) in 0.02 M TBS 0,02 with 3% hydrogen peroxide (Merck). Slides were counterstained with Mayer´s hematoxylin and mounted with Damar gum. Notch1 and Notch2 immunolabelings were evaluated under light microscope (Nikon) at X100 using an integrative eyepiece with a grid (X8, Zeiss No 2). Fields containing longitudinally sectioned glands were used (10 fields/animals) to quantify the total number of labeled epithelial cells and the distribution in gland compartments. The negative controls were obtained by omission of the primary antibody.

### 2.6 Immunofluorescence for Hes1

For Hes1 detection, non‐serial sections were prepared as described above and incubated with anti‐Hes1 rabbit polyclonal primary antibody (1 μL/mL, overnight, 4°C, MA532258, Invitrogen). After washing, sections were incubated with Alexa Fluor® 488‐conjugated goat anti‐rabbit IgG (20 μg/mL, 1 h, TA, Thermo Fisher). Nuclei were counterstained with DAPI (0.5 μg/mL), and slides were mounted in Mowiol®. Fluorescent images were captured using (Axioscope2, Zeiss software Zen Blue), and the distribution of Hes1+ cells was assessed according to the distribution in gland compartments. Ten fields from longitudinally sectioned glands were used to quantify the total number of labeled epithelial cells and the distribution in gland compartments. The negative controls were obtained by omission of the primary antibody.

### 2.7 Statistical Analyses

Data is presented as means ± standard deviation (SD). Comparisons between two groups were performed using unpaired Student's *t* test. For multiple group comparisons, one‐way ANOVA followed by Tukey's post hoc test was applied. Two‐way ANOVA was used to test the interaction of feeding conditions (S and EW) with the distribution of proliferative cells in the gastric gland in the different ages. Significance was set at *p* < 0.05. Graphs and analyses were conducted using GraphPad Prism 10.0.3 (GraphPad Software, San Diego, CA, USA).

## 3 Results

### 3.1 Early Weaning Alters Proliferative Cell Distribution and Anticipates Isthmus‐Restricted Cycling Profile

**Figure 2:**
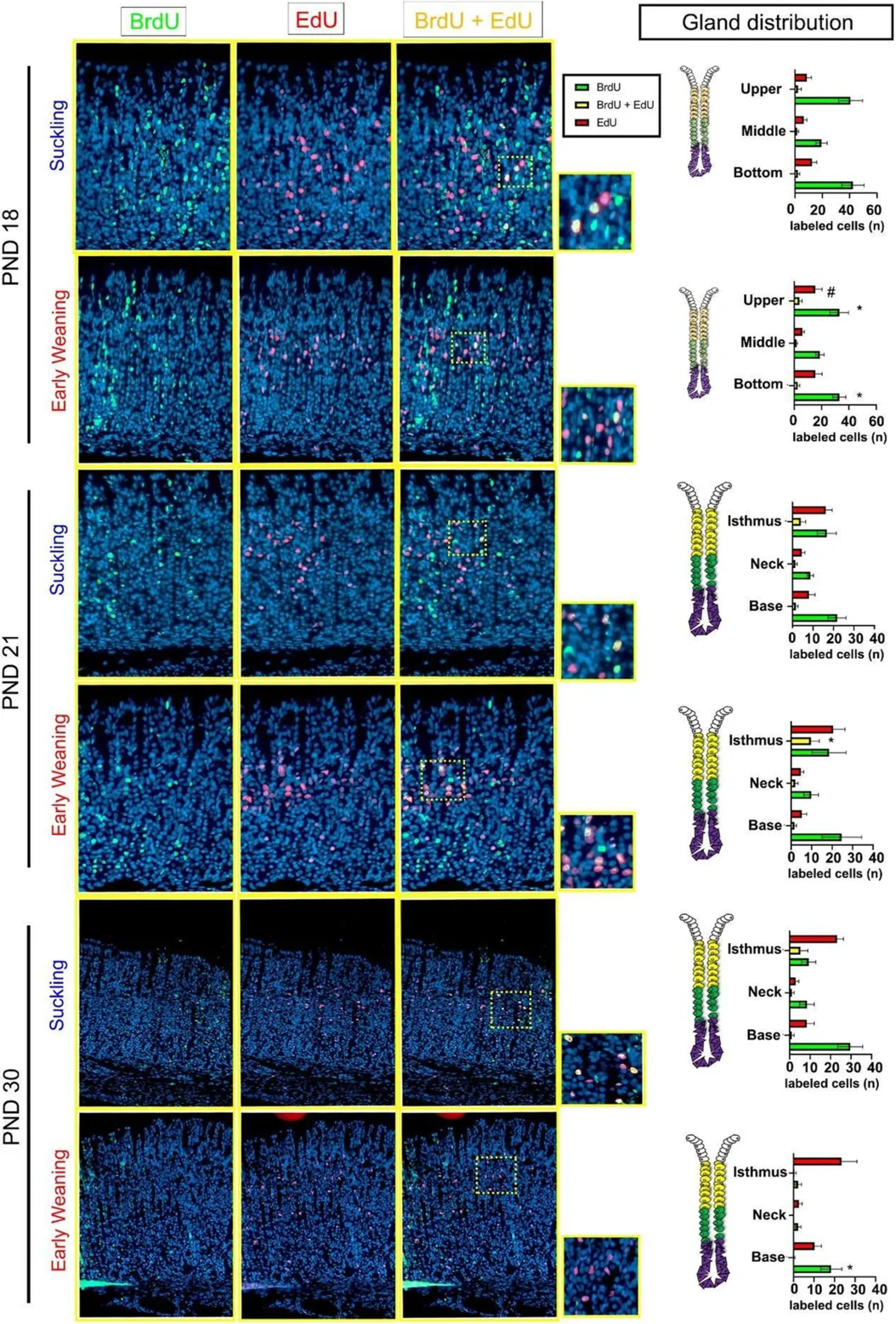
Distribution of BrdU+/EdU+ cells in the gastric corpus of suckling and early‐weaned rats. Representative photomicrographs under immunofluorescence for BrdU (488 nm – green), EdU (568 nm – red), and digital overlay for double labeling (BrdU+/EdU+ – yellow). Nuclei were counterstained with DAPI (blue). Images were acquired at original magnifications of ×400 (18 and 21 PND) and ×200 (30 PND). For each group at each age a detail is shown in the right. In the gland distribution column, the bar graphs represent the means ± SD of the number of labeled cells in each condition (*n* = 3 animals/group/age). At 18 PND days, the gland compartments were identified as upper, middle and base, and at 21 and 30 PND, they were identified as isthmus, neck and base. Two‐way ANOVA was used to test the interaction between feeding condition and gland region (*p* < 0.001), and *p < 0.05 compared to S group at the same region; ^#^ *p* < 0.05 compared to other regions within the same group.

Cell proliferation and migration were detected through BrdU and EdU labelings, and after chasing them, the BrdU+ cells represent the population that entered S phase during BrdU administration (PND 14 and 15); days later, BrdU was diluted between daughter‐cells and they migrated and differentiated. The EdU+ cells identified those that were in S phase during sample collection, and the BrdU+/EdU+ are the cells that were in S phase on PND 14 and 15 (BrdU+) and remained in the cell cycle or entered it *de novo* later on. As we started to track proliferative cells at 15 PND after BrdU administration, which was also EW onset, we observed that BrdU labeling is distributed along the whole gland (Figure 1). Then, to study EW effect on epithelial proliferation and dynamics during gastric postnatal development, we evaluated the different profiles of BrdU/EdU dual labeling at 18, 21 and 30 PND. On PND 18, we observed that EW induced a decrease of BrdU+ population when compared to suckling (S) animals (*p* < 0.0001) (Figure 2, Figure S1), especially in the upper and bottom areas of the gland (*p* < 0.05). The BrdU+/EdU+ cells were not affected by EW, although they were more concentrated in the upper gland (Figure 2, Figure S1). The EdU+ population (S‐phase cells) was not altered in number, but EW pushed them to the upper area of the gland (*p* < 0.05) (Figure 2, Figure S1). These results indicated that EW induced a precocious spatial restriction of the proliferative compartment in the gastric gland (Figure 2).

On PND 21, BrdU+ cells were observed mainly in the isthmus and base of the gland, similarly in S and EW animals (Figure 2, Figure S1). The BrdU+/EdU+ population was more concentrated in the isthmus and was increased by EW (*p* < 0.05). The EdU+ cells were also more numerous in this region, but they were not affected by EW (Figure 2, Figure S1). On PND 30, the number of BrdU+ cells was reduced by EW (*p* < 0.05), and such an effect was observed in the isthmus, neck and base, but they were more concentrated in the base (Figure 2, Figure S1). The BrdU+/EdU+ population was small in both groups, whereas EdU+ cells were highly distributed in the isthmus (Figure 2, Figure S1). After two‐way ANOVA we found that there was a significant interaction between feeding pattern and BrdU and EdU distribution (*p* < 0.0001), as EW affected the localization of the specific populations.

### 3.2 Early Weaning Modulates the Expression of Genes Associated With Gastric Proliferative Niche Regulation

**Figure 3:**
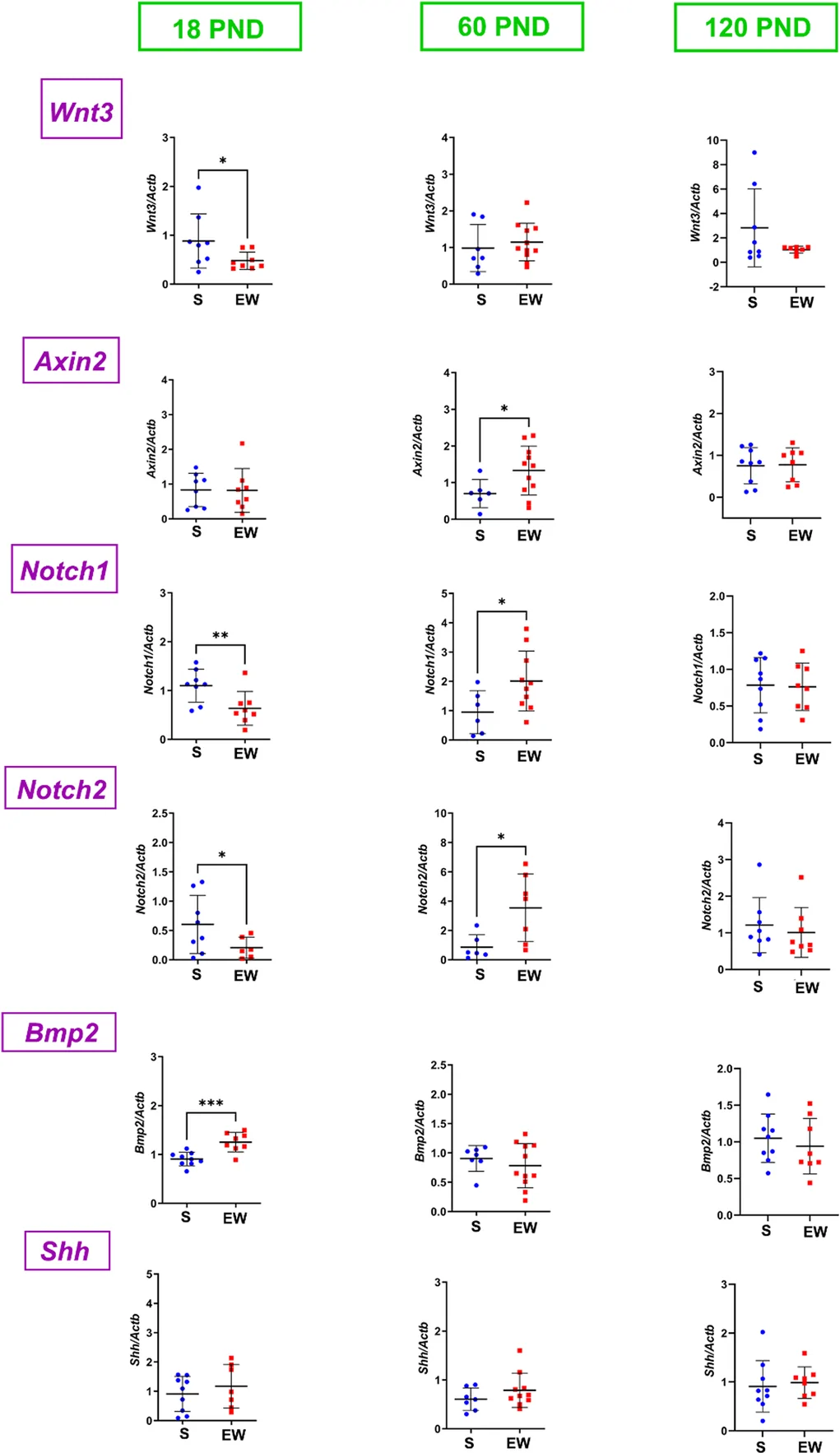
Expression of proliferative cell niche–related genes in the gastric corpus after early weaning. The relative expression of *Wnt3*, *Axin2*, *Notch1*, *Notch2*, *Bmp2* and *Shh* was analyzed after RT‐qPCR of gastric mucosa samples of S and EW animals at 18, 60 and 120 PND. *Actb* was used as internal control. Each dot represents one animal and lines indicate the means ± SD. (*n* = 5–6 animals/group/age). Results were analyzed by the Student *t* test. Samples were used as duplicates. *p < 0.05; **p < 0.01 and ***p < 0.001.

To investigate whether early weaning alters the molecular signaling involved in gastric stem cell niche regulation, we evaluated the mRNA expression of key components of the Wnt, BMP, Notch and Shh pathways in the gastric corpus at different postnatal stages (PND 18, 60, and 120). On PND 18, *Axin2* and *Shh* were not altered, whereas EW reduced the expression of *Wnt3*, *Notch1*, and *Notch2* compared to S controls (*p* < 0.05) (Figure 3), suggesting an early suppression of signals associated with stem cell maintenance and self‐renewal. In contrast, *Bmp2* expression was increased in the EW group at the same age (*p* < 0.05), consistent with a shift toward differentiation‐related signaling (Figure 3). These results indicated that EW may prematurely attenuate stemness‐associated transcriptional programs while enhancing cues involved in epithelial maturation.

On PND 60, however, a compensatory upregulation was observed: *Axin2*, *Notch1*, and *Notch2* transcript levels were significantly higher in EW animals than in age‐matched controls (*p* < 0.05) (Figure 3). This late increase may reflect a long‐term adaptation or rebound activation of proliferative signaling pathways in response to the early nutritional insult. No significant differences in gene expression were found between groups on PND 120 (Figure 3), suggesting that these molecular effects may be transient or stabilized by adulthood.

**Figure 4:**
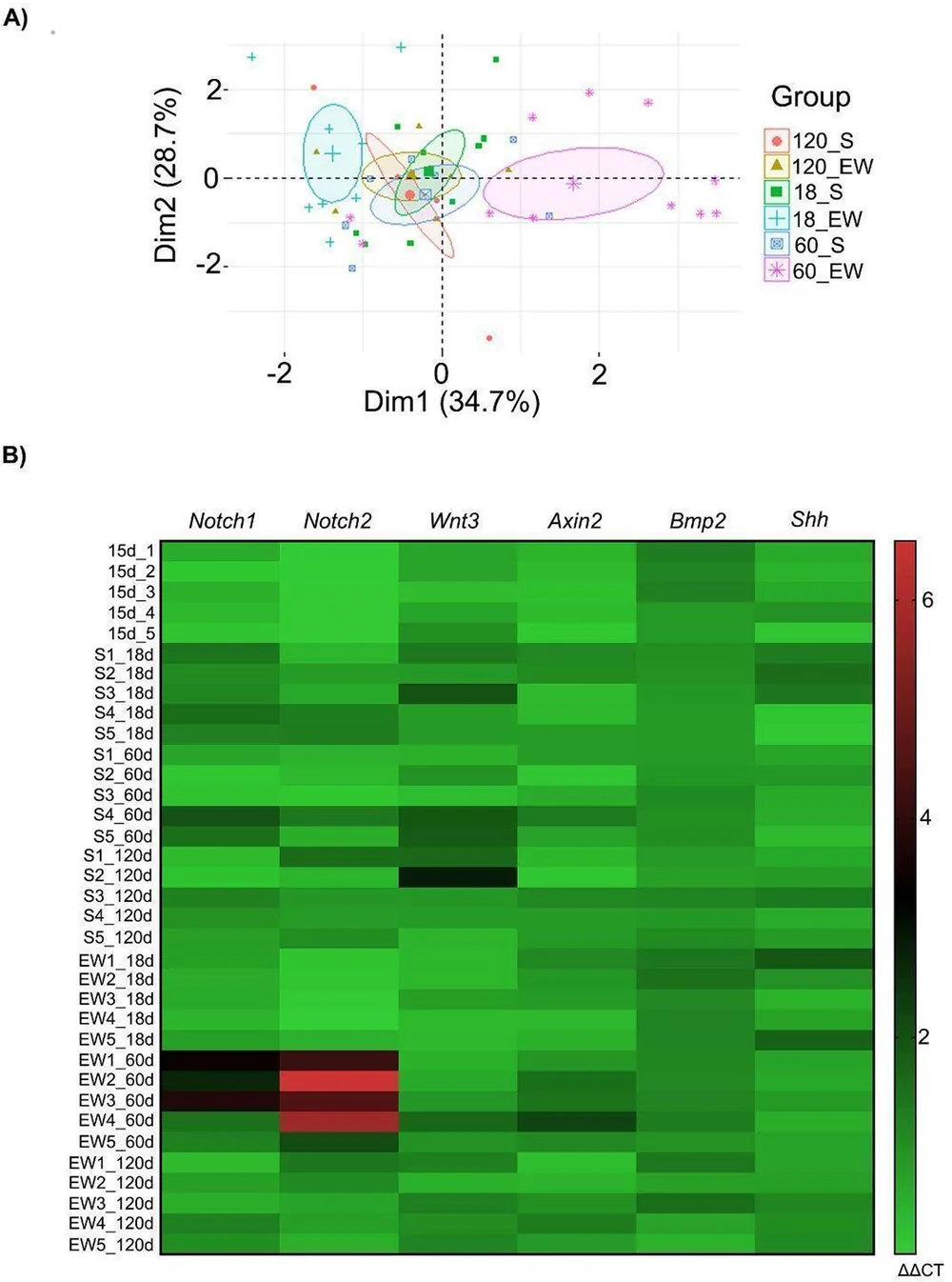
Heatmap summarizing the temporal modulation of gene expression induced by early weaning. (A) Principal component analysis (PCA)‐ the 2 dimensions represent the differential gene expression of S and EW rats at 18, 60 and 120 PND (clusters are identified by colors). X = principal component 1‐ Dim1 (34.7% of variance) and Y = principal component 2‐ Dim2 (28.7% of variance). (B) The heatmap depicts the fold‐change of expression of selected genes (*Notch1*, *Notch2, Wnt3*, *Axin2*, *Bmp2*, *and Shh*) in the gastric corpus of EW rats relative to age‐matched S controls. Each square represents the log_2_ fold‐change at a given postnatal day (PND 18, 60, or 120) (PND 15 group was added to allow the comparison among EW samples across ages). The red to green scale indicates high and low expression levels. Analyses made with *RStudio* software 1.0.153.

The principal component analysis (PCA) and the heatmap summarize the relative gene expression throughout aging (Figure 4) and highlighted the dynamic and time‐dependent impact of EW on key regulators of stem cell niches, mainly at 18 and 60 PND. Collectively, these findings support the hypothesis that nutritional signals during early life modulate the temporal dynamics of signaling pathways critical for gastric epithelial homeostasis and that EW induces an early downregulation followed by delayed upregulation of stemness‐associated genes.

### 3.3 In Silico Analysis Reveals Functional Specialization of Gastric Epithelial Cell Populations Through Distinct Signaling Profiles

To investigate the signaling profiles of functionally distinct gastric epithelial lineages, we performed an *in silico* analysis of gene expression data from GSE5018 (Ramsey et al. 2007), focusing on zymogenic and parietal cell populations isolated from the adult mouse gastric corpus. Processed microarray data were obtained from GEO and analyzed using Log_2_ transformation with a shared color scale, using KEGG pathway gene sets for Notch, BMP, Wnt, and SHH signaling pathways.

**Figure 5:**
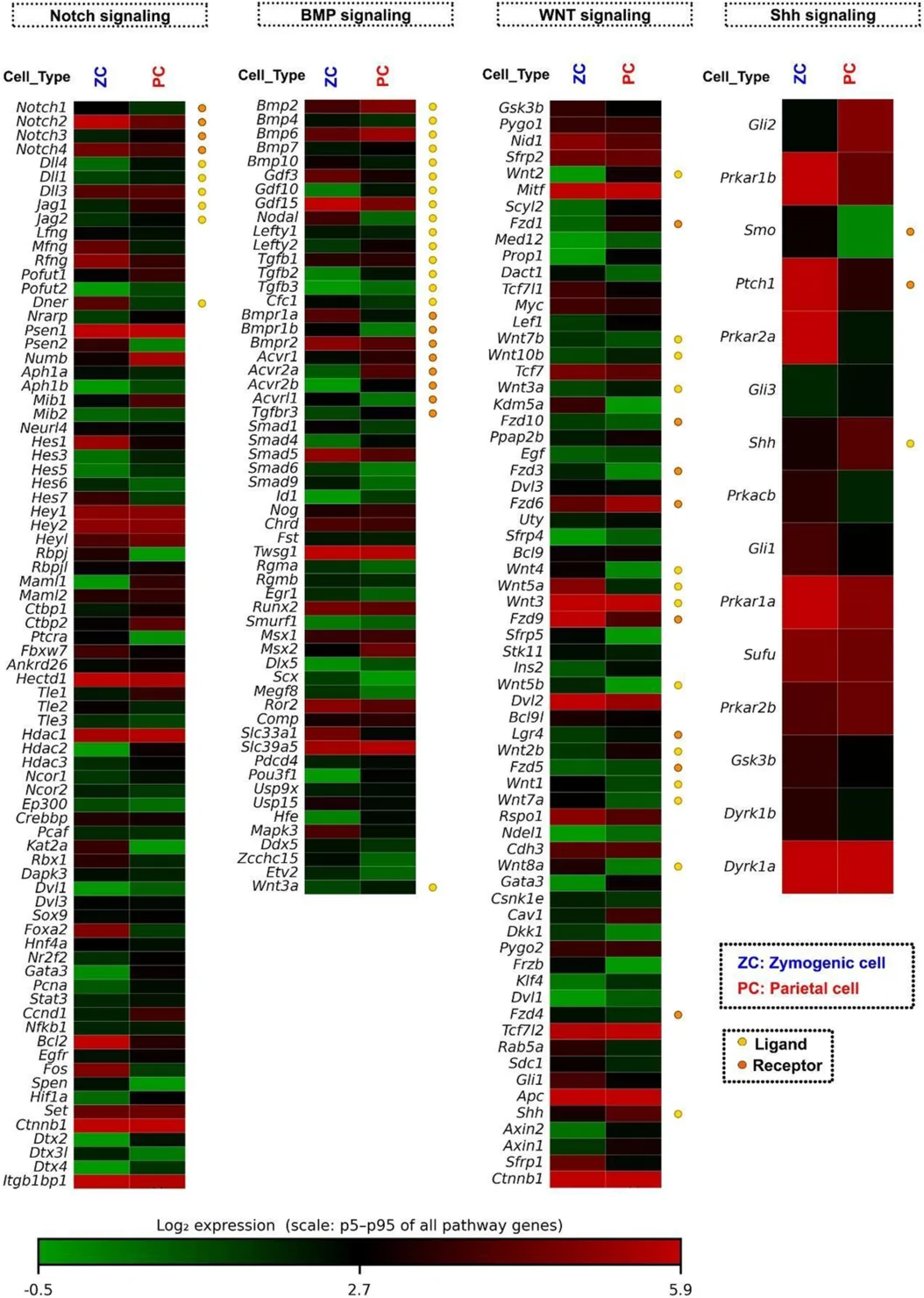
In silico studyof gene expression dataset and analysis parameters for zymogenic and parietal cells. Gene expression data were obtained from the public microarray dataset GSE5018, which contains transcriptomic profiles of isolated epithelial cell populations from the adult mouse stomach (zymogenic and parietal cells), considering Notch. BMP, Wnt and Shh pathways. Data analysis was performed using the R2: Genomics Analysis and Visualization Platform, applying the KEGG geneset collection and log_2_ z‐score transformation. In the heatmap, dark red represents higher expression levels, whereas green indicates lower expression. Circles mark ligands and receptors within each pathway: yellow for ligands and orange for receptors.

The expression profiles showed distinct signaling pathway activity between the two cell types (Figure 5, Table S1). In zymogenic cells, higher expression was observed for genes involved in canonical Wnt signaling, including *Axin2* (Δ = +2.1), *Lef1* (Δ = +1.3), *Tcf7l2* (Δ = +1.0), *Ctnnb1* (Δ = +1.1), and *Lgr4* (Δ = +1.0), consistent with a proliferative and self‐renewal‐promoting environment. Components of the Notch pathway were also differentially expressed between cell types: Notch ligands, including *Dll1*, *Dll4*, *Jag1*, and *Jag2*, showed higher expression in zymogenic cells, while Notch receptors *Notch1* and *Notch2*, along with the downstream effector *Hes1* and the co‐activator *Rbpj*, were more highly expressed in parietal cells (Figure 5, Table S1). This pattern suggests a paracrine mode in which zymogenic cells would send Notch signals to adjacent cell populations, rather than being the primary recipients of canonical Notch activation.

In contrast, parietal cells showed higher expression of genes related to BMP receptor signaling, including *Bmpr1a* (Δ = −1.9), *Bmpr1b* (Δ = −2.5), *Bmpr2* (Δ = −0.9), *Gdf15* (Δ = −2.1), and BMP‐responsive *Smad* mediators (*Smad1*, *Smad5*, *Smad6*, *Smad9*), pointing to active BMP‐mediated differentiation signaling in this cell type. Notably, BMP ligands *Bmp2*, *Bmp6*, and *Bmp7* showed higher expression in zymogenic cells, suggesting a paracrine BMP signaling axis in which ligands are produced by zymogenic cells and act on parietal cells through their cognate receptors. Within the SHH pathway, the ligand *Shh* and the transcription factor *Gli2* were more highly expressed in zymogenic cells, whereas the receptor *Ptch1* (Δ = −2.4) and the transcriptional repressor *Gli1* (Δ = −1.1) were enriched in parietal cells, again indicating a ligand‐in‐zymogenic, receptor‐in‐parietal paracrine architecture (Figure 5, Table S1).

These findings support the existence of cell‐type‐specific signaling niches within the gastric gland, in which zymogenic cells are embedded in a Wnt‐enriched and Notch ligand‐rich microenvironment, while parietal cells are characterized by active BMP receptor and SHH receptor signaling associated with terminal differentiation and trophic function. This compartmentalized architecture may play a critical role in mediating the long‐term effects of early‐life nutritional perturbations on epithelial dynamics.

### 3.4 Early Weaning and the Distribution of Notch1, Notch2 Receptors and Hes1 in the Gastric Gland

**Figure 6:**
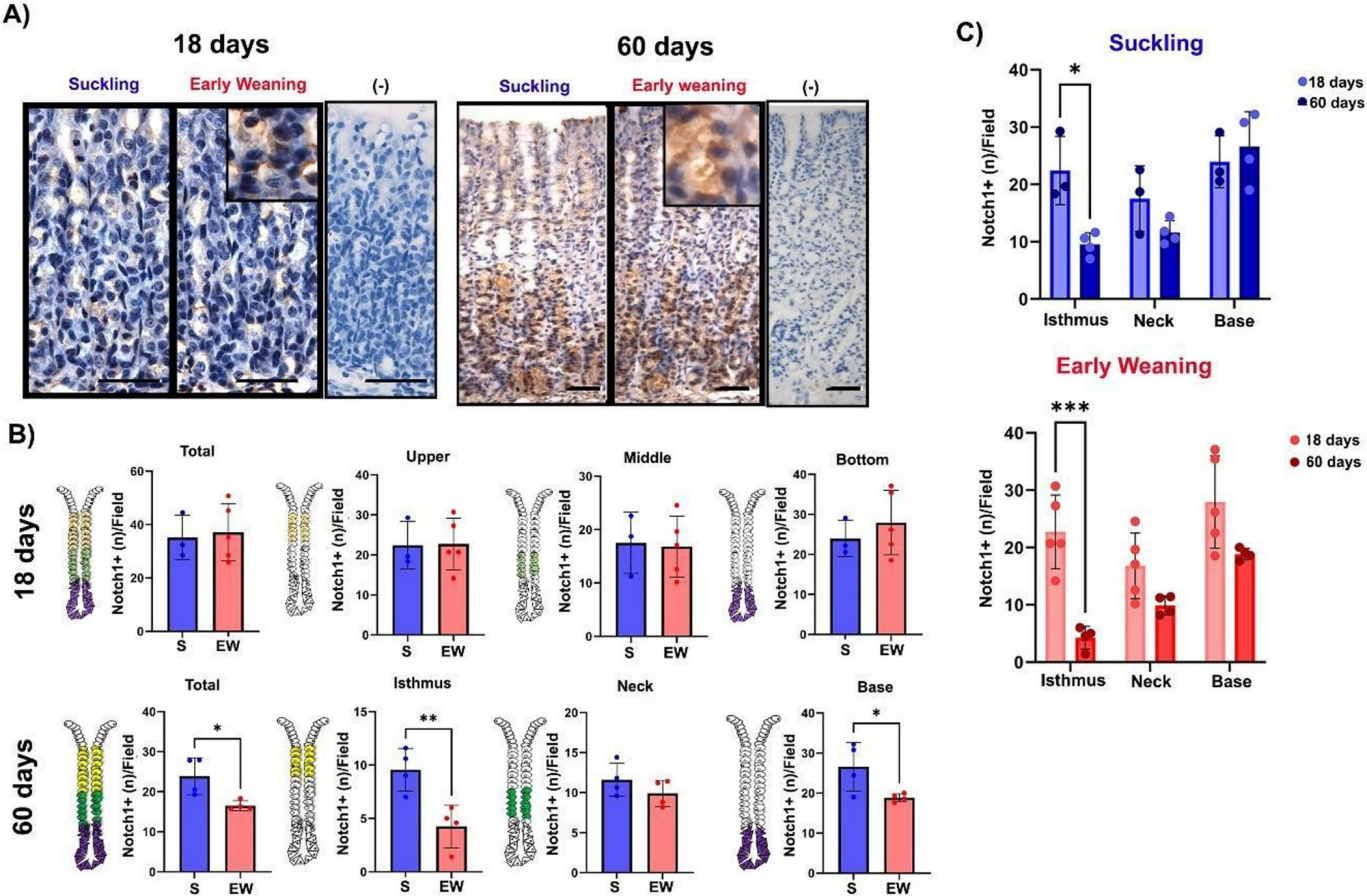
Spatial distribution of Notch1^+^ cells in the gastric corpus of rats at postnatal days 18 and 60. (A) Representative images of Notch1 distribution in S and EW rats, comparing the gastric mucosa at 18 and 60 PND. The immunolabeling was developed using DAB–H_2_O_2_ and counterstained with Mayer's hematoxylin. Negative controls are shown for each age. Scale bars = 50 μm Insets show the digital zoom to depict Notch1 immunolabeling. (B) Quantitative analyses compare the distribution of number of Notch1^+^ cells/field in the gland and across its regions in S and EW groups at each age. Statistical comparisons were performed using Student's *t* test. *p < 0.05; **p < 0.01; ***p < 0.0001. (C) Comparison of Notch1+ distribution among different ages, considering S and EW groups separately. Each dot represents one animal and lines represent means ± SD. (*n* = 3–5 per group). One‐way ANOVA followed by Tukey's post hoc test was used. *p < 0.05; **p < 0.01.

**Figure 7:**
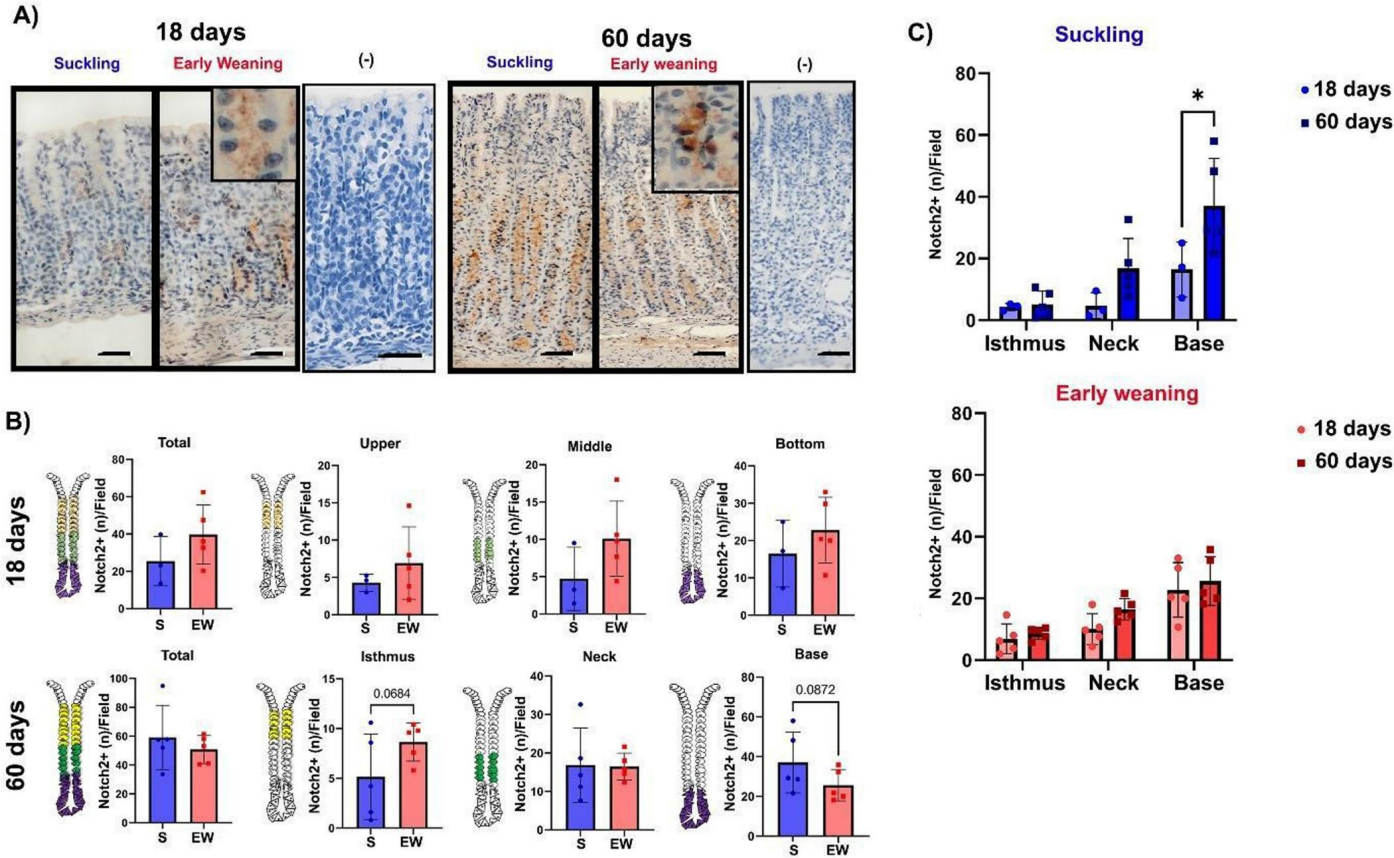
Spatial distribution of Notch2^+^ cells in the gastric corpus of rats at postnatal days 18 and 60. (A) Representative images of Notch2 distribution in S and EW rats, comparing the gastric mucosa at 18 and 60 PND. The immunolabeling was developed using DAB–H_2_O_2_ and counterstained with Mayer's hematoxylin. Negative controls are shown for each age. Scale bars = 50 μm Insets show the digital zoom to depict Notch2 immunolabeling. (B) Quantitative analyses compare the distribution of number of Notch2^+^ cells/field in the gland and across its regions in S and EW groups at each age. Statistical comparisons were performed using Student's *t* test. *p < 0.05; **p < 0.01; ***p < 0.0001. (C) Comparison of Notch2+ distribution among different ages, considering S and EW groups separately. Each dot represents one animal and lines represent means ± SD. (*n* = 3–5 per group). One‐way ANOVA followed by Tukey's post hoc test was used. *p < 0.05; **p < 0.01.

To evaluate whether early weaning alters the spatial distribution of Notch signaling components in the gastric corpus, we analyzed the number of immunolabeled cells/field for Notch1 and Notch2 in rats on PND 18 and 60 (Figures 6 and 7). At 18 PND, Notch1 was distributed along the gland (Figure 6A, C) and there was no difference between S and EW animals. At 60 PND, however, EW rats had a marked reduction of overall Notch1+ population (*p* < 0.05) when compared to S group, particularly in the isthmus (*p* < 0.01) and base (*p* < 0.05) (Figure 6B), suggesting a downregulation of Notch1 activity in the proliferative niches in this group. When the glands compartments were compared between ages in S and EW groups separately, we observed that from 18 to 60 PND a significant decrease was detected in the isthmus in both groups (*p* < 0.05 for S rats, and *p* < 0.001 for the EW ones) (Figure 6C) as the mucosa developed and grew.

When we analyzed Notch 2 distribution (Figure 7A) in S and EW groups and compared Notch2+ cells/field we did not detect significant differences between them at 18 PND (Figure 7B). At 60 PND EW increased Notch2 distribution in the isthmus (*p* = 0.0684) and reduced in the base (*p* = 0.0872) (Figure 7B), indicating a reorganization of Notch2‐positive cells within the glandular axis. This altered localization suggests that EW may shift Notch2 activity toward the upper compartment of the gland, potentially affecting the fate of progenitor cells and their lineage allocation. From 18 to 60 PND, we verified a larger Notch2+ cell population in the base of the gland in S rats, whereas EW did not induce a differential effect between ages (Figure 7C).

These findings demonstrate that Notch receptors respond differentially to early‐life nutritional cues, with Notch1 being globally downregulated and Notch2 exhibiting a compartment‐specific redistribution. Together, these changes imply a remodeling of Notch signaling architecture in the adult gastric epithelium because of early weaning.

To further evaluate the impact of EW on Notch pathway activity, we investigated the distribution of Hes1, a key transcriptional effector of canonical Notch signaling. Both at 18 and 60 PND, Hes1+ nuclei were distributed along the entire gland (Supplementary Figure S2), but when ages were compared, we detected an increase of Hes1+ population in the base (*p* < 0.0001 when 18 and 60 PND groups were compared for both S and EW conditions) (Supplementary Figure S2).

## 4 Discussion

In this study, we demonstrated that early weaning (EW) induces significant long‐lasting alterations in the organization of gastric epithelial proliferative niches, affecting not only the spatial distribution of dividing cells, but also the expression of key regulatory genes and the localization of signaling components involved in cell fate decisions. Together, these results support the hypothesis that nutritional cues, during the breastfeeding period, are critical for the postnatal maturation and compartmentalization of the gastric corpus epithelium.

Our data showed that EW accelerates the restriction of the proliferative zone to the isthmus, a pattern typically acquired after physiological weaning. This finding is consistent with previous studies from our group showing that EW promotes early glandular maturation, increased proliferative index, and accelerated emergence of the adult‐like glandular architecture (Mesquita da Silva et al. 2021). Notably, we now provided direct evidence that this maturation includes topographic reorganization of DNA synthesizing cells, as demonstrated by the redistribution of BrdU + /EdU+ cells. Moreover, our findings indicated that the acceleration of an adult‐like proliferative pattern in EW animals reflects not only increased proliferative activity, but also an early spatial reorganization of the cycling cell compartment. These results demonstrated that EW accelerates the establishment of an adult‐like proliferative profile in the gastric corpus, with the premature confinement of cycling cells to the isthmus region. This pattern is normally acquired only after physiological weaning, suggesting that maternal milk provides the trophic signals necessary to maintain a spread proliferative activity along the gland during the suckling period. Thus, the early withdrawal of these bioactive factors appears to anticipate the consolidation of the main proliferative niche, reinforcing previous studies showing that EW acts as a potent modulator of glandular maturation (Osaki et al. 2010; Mesquita da Silva et al. 2021; Rattes et al. 2025). These observations further support the idea that early nutritional cues shape the transition from the juvenile proliferative organization characterized by a broad distribution of cycling cells—to the adult architecture, in which proliferation becomes spatially restricted and functionally specialized.

Based on these observations, we suggest that two active proliferative niches in the isthmus and base contribute to gastric gland growth during the postnatal period. In adulthood, however, the isthmus becomes the dominant proliferative compartment, in line with prior reports (Adkins‐Threats et al. 2024; Burclaff et al. 2020; Han et al. 2019). Importantly, early weaning anticipated the restriction of proliferation to the isthmus, as observed on PND 18. Nonetheless, at PND 30, EW animals showed a reduction in the total BrdU^+^ population, with the remaining labeled cells concentrated in the gland base, suggesting a redistribution rather than an expansion of proliferative activity in this compartment. In the adult gastric mucosa, previous studies from our group reported an expansion of the zymogenic cell population following early weaning (Teles Silva et al. 2020; Zulian et al. 2017), which may reflect a long‐term consequence of the early reorganization of proliferative niches documented here.

At the molecular level, our results indicated that early weaning modulates the Notch signaling pathway in a developmental stage–dependent manner. On PND 18, EW reduced the expression of *Notch1*, *Notch2* and the downstream effector *Hes1*. This pattern suggests an early decrease in canonical Notch activity during a phase in which proliferative domains are still being defined. These findings are consistent with the established role of Notch in supporting progenitor proliferation and regulating epithelial differentiation in the gastric antrum and corpus, where Notch1 and Notch2 function as the main receptors controlling progenitor maintenance (Demitrack et al. 2015; Demitrack et al. 2017; Gifford et al. 2017).

In addition to transcriptional changes, EW altered the spatial localization of Notch pathway components, Notch1 and Notch2, especially at 60 PND, and our observations corroborate the studies that showed that Notch contributes to regional organization of gastric epithelial niches (Han et al. 2019; Willet and Mills 2016), also in different cellular functions (McKimpson et al. 2024). In our model, it suggests that EW did not uniformly suppress or enhance Notch activity, but instead, it modified its temporal progression during postnatal development. We can suggest that nutritional disruption during the breastfeeding period can influence the spatial refinement of these signaling domains.

Taken together, these findings support the idea that early weaning influences the maturation of gastric proliferative niches by altering both the magnitude and the spatial distribution of Notch pathway activity. Because Notch is a key regulator of progenitor maintenance and differentiation in the gastrointestinal tract (Kim and Shivdasani 2011), changes in its developmental patterning may contribute to the earlier establishment of the isthmus‐restricted proliferative profile observed in EW pups as well as to the persistence of modified niche characteristics later in life. This developmental perspective highlights how early‐life nutritional conditions can shape the signaling landscape that guides gastric epithelial maturation.

The *in silico* analyses further reinforced the definition of a spatially organized and paracrine signaling gradient in the gastric epithelium. The current analysis of the GSE5018 dataset with raw Log_2_ expression indicated that zymogenic cells displayed higher levels of canonical Wnt target genes (*Axin2*, *Lef1*, *Ctnnb1*, *Lgr4*) and Notch pathway ligands (*Dll1*, *Dll4*, *Jag1*, *Jag2*), while parietal cells showed enrichment for BMP receptors (*Bmpr1a*, *Bmpr1b*, *Gdf15*), BMP‐responsive Smad mediators (*Smad1*, *Smad5*, *Smad9*), and the SHH receptor *Ptch1*. Importantly, BMP ligands (*Bmp2*, *Bmp6*) and the SHH ligand *Shh* were more highly expressed in zymogenic cells, whereas their cognate receptors were enriched in parietal cells. Similarly, Notch receptors *Notch1* and *Notch2*, along with the effector *Hes1*, were more highly transcripted in parietal cells, while zymogenic cells expressed higher levels of Notch ligands. Together, these patterns are consistent with a paracrine signaling model in which zymogenic cells serve as the source of BMP, SHH, and Notch signals that are received and transduced by adjacent parietal cells. These findings support a model in which distinct epithelial lineages rely on complementary, region‐specific signaling profiles—with zymogenic cells sustaining a renewal‐associated transcriptional state through Wnt activity, while parietal cells respond to differentiation and trophic cues delivered in part by their neighbors. Disruption of early‐life nutritional cues, as observed after early weaning, may shift these paracrine balances and contribute to the persistent changes in niche organization described in this study.

Taken together, our findings expand on previous studies by demonstrating that early nutritional withdrawal affects both structural and molecular features of the gastric niche, with persistent consequences on epithelial organization and potential implications for gastric function and disease susceptibility later in life.

## Author Contributions

**Isadora Campos Rattes:** conceptualization, investigation, writing – original draft, methodology, validation, visualization, writing – review and editing, formal analysis, data curation. **Marcos Aurélio Santos da Costa:** methodology, writing – review and editing, validation, investigation. **Viviane Rosa de Oliveira:** investigation, methodology, validation, writing – review and editing, formal analysis. **Patrícia Gama:** conceptualization, investigation, funding acquisition, writing – original draft, visualization, writing – review and editing, formal analysis, project administration, data curation, supervision, resources.

## Conflicts of Interest

The authors declare no conflicts of interest.

## Supporting information


Supporting File

## Data Availability

The data that support the findings of this study are openly available at: https://drive.google.com/drive/folders/1e-VKq3O9YHtnp0s0-5FViEDbxncznIxK?usp=sharing. The planning for the whole project is available at Patricia Gama, Isadora Campos Rattes, Aline da Costa Vasques, Israel Tojal da Silva, Marcos Aurélio Santos da Costa, Viviane Rosa de Oliveira. (2025). “Characterization of cell proliferation behavior in the rat gastric mucosa: immediate and late effects of early weaning” [Data Management Plan]. DMP Tool. https://doi.org/10.48321/D1JC7C.
