# Supplementary material for: Early‐Life Nutritional Disruption Reshapes Gastric Epithelial Development Through Coordinated Changes in the Dynamics and Signaling of Proliferative Niches
Source: J Cell Physiol. 2026 Sep 25;241(9):e70234. doi: 10.1002/jcp.70234 (PMC13613554; doi:10.1002/jcp.70234)
Supplement: Supplementary file 1 [file JCP-241-0-s001.docx]

**Early-Life Nutritional Disruption Reshapes Gastric Epithelial Development Through Coordinated Changes in the Dynamics and Signaling of Proliferative Niches**

Isadora Campos Rattes, Marcos Aurélio Santos da Costa, Viviane Rosa de Oliveira, Patrícia Gama

Department of Cell and Developmental Biology, Institute of Biomedical Sciences, University of Sao Paulo

**Supplementary Figure 1**. Representation of distribution of BrdU+, EdU+, and BrdU-EdU+ populations at 18, 21, and 30 PND in both S and EW groups. After obtaining the number of labeled cells within each glandular region, statistical comparisons were conducted between groups (S vs. EW). The bar graphs display the means ± SD of labeled cells for each labeling condition and group (n = 3). Results were evaluated by Two-Way ANOVA. **p* < 0.05, ***p* < 0.01, ****p* < 0.001, *****p* < 0.0001.

**Supplementary Figure 2. Spatial distribution of Hes1⁺ cells in the gastric corpus of rats at postnatal days 18 and 60.**

(A) Regional distribution of Hes1⁺ nuclei in suckling (S) and early-weaned (EW) rats at PND 18 and PND 60. (B) Age-related comparison of Hes1⁺ cell distribution within S and EW groups. Each dot represents an individual animal; horizontal bars indicate means ± SD. Statistical analyses were performed using Student’s *t*-test and two-way ANOVA. **** *p*< 0.0001 (n = 4–5 per group).

**Supplementary Figure 1**

**Supplementary Figure 2**

| **Supplementary Table 1 — GSE5018 Gene Expression: Parietal vs. Zymogenic Cells** | | | | | |
| --- | --- | --- | --- | --- | --- |
| *Source: Ramsey et al. (2007). Affymetrix Mouse Genome 430 2.0. GEO accession: GSE5018 (GSM113346 = Parietal; GSM113347 = Zymogenic). Log₂ values from dChip processing.* | | | | | |
| **Pathway** | **Gene** | **Probe ID (GPL1261)** | **Annotation** | **Parietal Log₂** | **Zymogenic Log₂** |
| **Canonical WNT Signaling** | | | | | |
| Canonical WNT Signaling | *Gsk3b* | 1460700_at | — | 3,5154 | 2,7749 |
| Canonical WNT Signaling | *Pygo1* | 1416435_at | — | 3,5686 | 3,6300 |
| Canonical WNT Signaling | *Nid1* | 1416382_at | — | 4,9529 | 4,1660 |
| Canonical WNT Signaling | *Sfrp2* | 1449353_at | — | 4,4929 | 4,4222 |
| Canonical WNT Signaling | *Wnt2* | 1422681_at | L | -9,9658 | 2,9789 |
| Canonical WNT Signaling | *Mitf* | 1416556_at | — | 6,1049 | 5,9249 |
| Canonical WNT Signaling | *Scyl2* | 1419731_at | — | 0,5116 | 2,6703 |
| Canonical WNT Signaling | *Fzd1* | 1425558_at | R | 0,5821 | 3,1829 |
| Canonical WNT Signaling | *Med12* | 1454483_at | — | -20,1176 | 0,7925 |
| Canonical WNT Signaling | *Prop1* | 1421494_at | — | -1,3792 | 2,6014 |
| Canonical WNT Signaling | *Dact1* | 1430518_at | — | 2,3875 | 0,6405 |
| Canonical WNT Signaling | *Tcf7l1* | 1450416_at | — | 3,7370 | 2,8825 |
| Canonical WNT Signaling | *Myc* | 1424942_a_at | — | 3,7671 | 3,4064 |
| Canonical WNT Signaling | *Lef1* | 1421431_at | — | 1,4964 | 2,7775 |
| Canonical WNT Signaling | *Wnt7b* | 1419603_at | L | 1,6878 | 1,0667 |
| Canonical WNT Signaling | *Wnt10b* | 1425984_at | L | 1,2628 | 2,0395 |
| Canonical WNT Signaling | *Tcf7* | 1426903_at | — | 4,6307 | 4,2644 |
| Canonical WNT Signaling | *Wnt3a* | 1416003_at | L | 1,2369 | 2,1146 |
| Canonical WNT Signaling | *Kdm5a* | 1427392_at | — | 3,6034 | -0,5377 |
| Canonical WNT Signaling | *Fzd10* | 1450910_at | R | 1,4459 | 0,8127 |
| Canonical WNT Signaling | *Ppap2b* | 1415730_at | — | 2,1685 | 2,9772 |
| Canonical WNT Signaling | *Egf* | 1416071_at | — | 0,7242 | 1,2034 |
| Canonical WNT Signaling | *Fzd3* | 1438083_at | R | 1,9982 | -0,1673 |
| Canonical WNT Signaling | *Dvl3* | 1423063_at | — | 2,5896 | 2,4624 |
| Canonical WNT Signaling | *Fzd6* | 1416498_at | R | 4,2867 | 5,2589 |
| Canonical WNT Signaling | *Uty* | 1437287_at | — | 2,0501 | 2,4397 |
| Canonical WNT Signaling | *Sfrp4* | 1448834_at | — | -9,9658 | 0,7603 |
| Canonical WNT Signaling | *Bcl9* | 1437748_at | — | 2,8713 | 2,9851 |
| Canonical WNT Signaling | *Wnt4* | 1416529_at | L | 2,8615 | -0,1248 |
| Canonical WNT Signaling | *Wnt5a* | 1415899_at | L | 4,9157 | 1,8423 |
| Canonical WNT Signaling | *Wnt3* | 1416128_at | L | 10,1531 | 6,3323 |
| Canonical WNT Signaling | *Fzd9* | 1423277_at | R | 6,2069 | 3,9749 |
| Canonical WNT Signaling | *Sfrp5* | 1421573_at | — | 2,5155 | -2,2881 |
| Canonical WNT Signaling | *Stk11* | 1419276_at | — | 2,3808 | 2,0676 |
| Canonical WNT Signaling | *Ins2* | 1415898_at | — | 0,8507 | 2,4176 |
| Canonical WNT Signaling | *Wnt5b* | 1436530_at | L | 1,9202 | -9,9658 |
| Canonical WNT Signaling | *Dvl2* | 1416528_at | — | 7,1733 | 5,2711 |
| Canonical WNT Signaling | *Bcl9l* | 1451743_at | — | 3,1554 | 2,8189 |
| Canonical WNT Signaling | *Lgr4* | 1449403_at | R | 1,4121 | 2,4163 |
| Canonical WNT Signaling | *Wnt2b* | 1416553_at | L | 1,5679 | 3,1174 |
| Canonical WNT Signaling | *Fzd5* | 1416071_at | R | 0,7242 | 1,2034 |
| Canonical WNT Signaling | *Wnt1* | 1449081_at | L | 2,7349 | 1,2816 |
| Canonical WNT Signaling | *Wnt7a* | 1422884_at | L | 2,7518 | 0,8568 |
| Canonical WNT Signaling | *Rspo1* | 1452579_at | — | 4,9640 | 4,0581 |
| Canonical WNT Signaling | *Ndel1* | 1420200_at | — | -4,0040 | 0,4565 |
| Canonical WNT Signaling | *Cdh3* | 1450944_at | — | 4,0939 | 4,1030 |
| Canonical WNT Signaling | *Wnt8a* | 1421712_at | L | 3,1751 | 0,0840 |
| Canonical WNT Signaling | *Gata3* | 1416467_at | — | -0,1993 | 2,8946 |
| Canonical WNT Signaling | *Csnk1e* | 1416752_at | — | 2,0396 | 1,5812 |
| Canonical WNT Signaling | *Cav1* | 1415840_at | — | 2,0277 | 3,7517 |
| Canonical WNT Signaling | *Dkk1* | 1418120_at | — | 1,6097 | -0,0226 |
| Canonical WNT Signaling | *Pygo2* | 1416435_at | — | 3,5686 | 3,6300 |
| Canonical WNT Signaling | *Frzb* | 1421573_at | — | 2,5155 | -2,2881 |
| Canonical WNT Signaling | *Klf4* | 1421539_at | — | 0,2608 | 1,7493 |
| Canonical WNT Signaling | *Dvl1* | 1416527_at | — | -1,4048 | 0,7731 |
| Canonical WNT Signaling | *Fzd4* | 1417796_at | R | 2,3549 | 1,7963 |
| Canonical WNT Signaling | *Tcf7l2* | 1416951_a_at | — | 5,6622 | 6,6292 |
| Canonical WNT Signaling | *Rab5a* | 1415826_at | — | 3,3820 | 1,9308 |
| Canonical WNT Signaling | *Sdc1* | 1415800_at | — | 2,8838 | 1,9045 |
| Canonical WNT Signaling | *Gli1* | 1417182_at | — | 3,8101 | 2,7560 |
| Canonical WNT Signaling | *Apc* | 1448292_at | — | 6,8982 | 6,0307 |
| Canonical WNT Signaling | *Shh* | 1451553_at | L | 3,0954 | 4,1042 |
| Canonical WNT Signaling | *Axin2* | 1415978_at | — | 0,3715 | 2,4963 |
| Canonical WNT Signaling | *Axin1* | 1448984_at | — | 1,6367 | 3,0950 |
| Canonical WNT Signaling | *Sfrp1* | 1415993_at | — | 4,3534 | 2,5679 |
| Canonical WNT Signaling | *Ctnnb1* | 1426974_at | — | 6,0214 | 7,1565 |
| **Notch Signaling** | | | | | |
| Notch Signaling | *Notch1* | 1416569_at | R | 2,7742 | 1,6668 |
| Notch Signaling | *Notch2* | 1422833_at | R | 6,5031 | 4,3538 |
| Notch Signaling | *Notch3* | 1422835_at | R | 2,0172 | 2,8506 |
| Notch Signaling | *Notch4* | 1452206_at | R | 4,5596 | 3,9504 |
| Notch Signaling | *Dll4* | 1426438_at | L | 0,5068 | 2,3398 |
| Notch Signaling | *Dll1* | 1422890_at | L | 1,3298 | 2,1394 |
| Notch Signaling | *Dll3* | 1450944_at | L | 4,0939 | 4,1030 |
| Notch Signaling | *Jag1* | 1420496_at | L | 1,9596 | 3,4839 |
| Notch Signaling | *Jag2* | 1422434_a_at | L | 1,6766 | 2,5210 |
| Notch Signaling | *Lfng* | 1417832_at | — | 2,5767 | 2,3453 |
| Notch Signaling | *Mfng* | 1423127_at | — | 4,3589 | 2,0371 |
| Notch Signaling | *Rfng* | 1416508_at | — | 5,0364 | 3,5925 |
| Notch Signaling | *Pofut1* | 1451219_at | — | 2,7219 | 3,6471 |
| Notch Signaling | *Pofut2* | 1427174_at | — | -0,6286 | 1,2315 |
| Notch Signaling | *Dner* | 1420952_at | L | 4,1698 | 1,5552 |
| Notch Signaling | *Nrarp* | 1424044_at | — | 1,4293 | 2,8158 |
| Notch Signaling | *Psen1* | 1416556_at | — | 6,1049 | 5,9249 |
| Notch Signaling | *Psen2* | 1416558_at | — | 3,4793 | -0,0056 |
| Notch Signaling | *Numb* | 1421071_at | — | 2,9583 | 5,4666 |
| Notch Signaling | *Aph1a* | 1428023_at | — | 2,4054 | 1,9186 |
| Notch Signaling | *Aph1b* | 1451199_at | — | -1,2753 | 1,0910 |
| Notch Signaling | *Mib1* | 1425765_at | — | 2,5589 | 3,9018 |
| Notch Signaling | *Mib2* | 1416561_at | — | 0,5524 | 1,2118 |
| Notch Signaling | *Neurl4* | 1450789_at | — | 2,8876 | 2,5396 |
| Notch Signaling | *Hes1* | 1422880_at | — | 5,2976 | 3,0568 |
| Notch Signaling | *Hes3* | 1449720_at | — | 0,3623 | 2,0176 |
| Notch Signaling | *Hes5* | 1421539_at | — | 0,2608 | 1,7493 |
| Notch Signaling | *Hes6* | 1416380_at | — | 1,8814 | 0,7207 |
| Notch Signaling | *Hes7* | 1451198_at | — | 3,5657 | 1,4738 |
| Notch Signaling | *Hey1* | 1416712_at | — | 5,0297 | 4,9829 |
| Notch Signaling | *Hey2* | 1416712_at | — | 5,0297 | 4,9829 |
| Notch Signaling | *Heyl* | 1427069_at | — | 3,9099 | 4,4766 |
| Notch Signaling | *Rbpj* | 1423423_at | — | 3,2789 | -2,4859 |
| Notch Signaling | *Rbpjl* | 1427423_at | — | 2,8328 | 3,0321 |
| Notch Signaling | *Maml1* | 1432519_at | — | -1,5160 | 3,5226 |
| Notch Signaling | *Maml2* | 1448875_at | — | 3,3429 | 3,5375 |
| Notch Signaling | *Ctbp1* | 1415730_at | — | 2,1685 | 2,9772 |
| Notch Signaling | *Ctbp2* | 1416006_at | — | 2,8272 | 4,2187 |
| Notch Signaling | *Ptcra* | 1421285_at | — | 2,7156 | -0,3366 |
| Notch Signaling | *Fbxw7* | 1450416_at | — | 3,7370 | 2,8825 |
| Notch Signaling | *Ankrd26* | 1452340_at | — | 2,4755 | 2,8981 |
| Notch Signaling | *Hectd1* | 1437148_at | — | 8,1859 | 5,6430 |
| Notch Signaling | *Tle1* | 1454711_at | — | 2,2197 | 3,5147 |
| Notch Signaling | *Tle2* | 1415800_at | — | 2,8838 | 1,9045 |
| Notch Signaling | *Tle3* | 1415801_at | — | 1,8067 | 1,2924 |
| Notch Signaling | *Hdac1* | 1415727_at | — | 5,4741 | 5,7001 |
| Notch Signaling | *Hdac2* | 1415728_at | — | -0,5854 | 2,9618 |
| Notch Signaling | *Hdac3* | 1425588_at | — | 1,7574 | 2,6070 |
| Notch Signaling | *Ncor1* | 1429517_at | — | 1,6120 | 2,3260 |
| Notch Signaling | *Ncor2* | 1416752_at | — | 2,0396 | 1,5812 |
| Notch Signaling | *Ep300* | 1425556_at | — | 1,1748 | 0,4367 |
| Notch Signaling | *Crebbp* | 1428428_at | — | 3,2732 | 3,0423 |
| Notch Signaling | *Pcaf* | 1425831_at | — | 1,8487 | 1,7627 |
| Notch Signaling | *Kat2a* | 1427392_at | — | 3,6034 | -0,5377 |
| Notch Signaling | *Rbx1* | 1415826_at | — | 3,3820 | 1,9308 |
| Notch Signaling | *Dapk3* | 1419276_at | — | 2,3808 | 2,0676 |
| Notch Signaling | *Dvl1* | 1416527_at | — | -1,4048 | 0,7731 |
| Notch Signaling | *Dvl3* | 1423063_at | — | 2,5896 | 2,4624 |
| Notch Signaling | *Sox9* | 1422820_at | — | 2,5199 | 2,7673 |
| Notch Signaling | *Foxa2* | 1423100_at | — | 4,8011 | 1,5232 |
| Notch Signaling | *Hnf4a* | 1421958_at | — | 2,6501 | 2,3634 |
| Notch Signaling | *Nr2f2* | 1422149_at | — | 1,7422 | 2,7916 |
| Notch Signaling | *Gata3* | 1416467_at | — | -0,1993 | 2,8946 |
| Notch Signaling | *Pcna* | 1415898_at | — | 0,8507 | 2,4176 |
| Notch Signaling | *Stat3* | 1417430_at | — | 1,9067 | 2,2693 |
| Notch Signaling | *Ccnd1* | 1415840_at | — | 2,0277 | 3,7517 |
| Notch Signaling | *Nfkb1* | 1418345_at | — | 1,9416 | 2,1104 |
| Notch Signaling | *Bcl2* | 1416041_at | — | 7,8720 | 3,3469 |
| Notch Signaling | *Egfr* | 1417072_at | — | 2,3064 | 2,8988 |
| Notch Signaling | *Fos* | 1423100_at | — | 4,8011 | 1,5232 |
| Notch Signaling | *Spen* | 1449532_at | — | 2,3627 | -2,7664 |
| Notch Signaling | *Hif1a* | 1419731_at | — | 0,5116 | 2,6703 |
| Notch Signaling | *Set* | 1415958_at | — | 4,3886 | 4,4719 |
| Notch Signaling | *Ctnnb1* | 1426974_at | — | 6,0214 | 7,1565 |
| Notch Signaling | *Dtx2* | 1434738_at | — | -0,6129 | 2,2834 |
| Notch Signaling | *Dtx3l* | 1428013_at | — | 2,0613 | 0,1708 |
| Notch Signaling | *Dtx4* | 1434739_at | — | -2,3286 | 1,6701 |
| Notch Signaling | *Itgb1bp1* | 1416531_at | — | 12,0919 | 5,6611 |
| **BMP Signaling** | | | | | |
| BMP Signaling | *Bmp2* | 1421029_a_at | L | 3,8005 | 4,8363 |
| BMP Signaling | *Bmp4* | 1449212_at | L | 2,3744 | 1,7587 |
| BMP Signaling | *Bmp6* | 1416498_at | L | 4,2867 | 5,2589 |
| BMP Signaling | *Bmp7* | 1421112_at | L | 2,2357 | 2,8078 |
| BMP Signaling | *Bmp10* | 1418828_at | L | 2,9741 | 2,1293 |
| BMP Signaling | *Gdf3* | 1421810_at | L | 4,3466 | 3,1300 |
| BMP Signaling | *Gdf10* | 1449602_at | L | 0,1096 | 2,2417 |
| BMP Signaling | *Gdf15* | 1418119_at | L | 6,7700 | 4,7153 |
| BMP Signaling | *Nodal* | 1450877_at | L | 3,7135 | 0,5406 |
| BMP Signaling | *Lefty1* | 1419082_at | L | 2,2414 | 2,0297 |
| BMP Signaling | *Lefty2* | 1419085_at | L | 1,5544 | 2,9292 |
| BMP Signaling | *Tgfb1* | 1426835_at | L | 3,3952 | 3,4681 |
| BMP Signaling | *Tgfb2* | 1416855_at | L | 0,0201 | 2,2939 |
| BMP Signaling | *Tgfb3* | 1420200_at | L | -4,0040 | 0,4565 |
| BMP Signaling | *Cfc1* | 1418944_at | L | 2,4995 | 1,6017 |
| BMP Signaling | *Bmpr1a* | 1449004_at | R | 4,1247 | 2,2726 |
| BMP Signaling | *Bmpr1b* | 1421283_at | R | 2,5979 | 0,0806 |
| BMP Signaling | *Bmpr2* | 1452579_at | R | 4,9640 | 4,0581 |
| BMP Signaling | *Acvr1* | 1415992_at | R | 2,4893 | 3,4274 |
| BMP Signaling | *Acvr2a* | 1448985_at | R | 0,8812 | 4,0013 |
| BMP Signaling | *Acvr2b* | 1421284_at | R | -0,5199 | 2,6674 |
| BMP Signaling | *Acvrl1* | 1420998_at | R | 2,6296 | 0,2947 |
| BMP Signaling | *Tgfbr3* | 1439569_at | R | 1,2451 | 2,6637 |
| BMP Signaling | *Smad1* | 1418174_at | — | 2,8467 | 1,4206 |
| BMP Signaling | *Smad4* | 1449029_at | — | 0,4110 | 2,4693 |
| BMP Signaling | *Smad5* | 1416695_at | — | 5,0900 | 4,0691 |
| BMP Signaling | *Smad6* | 1416754_at | — | 1,5758 | 0,0900 |
| BMP Signaling | *Smad9* | 1418396_at | — | 2,2198 | 0,4941 |
| BMP Signaling | *Id1* | 1418459_at | — | -1,8514 | 1,5029 |
| BMP Signaling | *Nog* | 1417567_at | — | 3,2023 | 3,5625 |
| BMP Signaling | *Chrd* | 1422394_at | — | 3,9507 | 3,7581 |
| BMP Signaling | *Fst* | 1419082_at | — | 2,2414 | 2,0297 |
| BMP Signaling | *Twsg1* | 1424174_at | — | 7,7430 | 5,7123 |
| BMP Signaling | *Rgma* | 1448964_at | — | 1,7290 | 0,6549 |
| BMP Signaling | *Rgmb* | 1451556_a_at | — | 2,0737 | 1,8608 |
| BMP Signaling | *Egr1* | 1421061_at | — | 1,8493 | 0,7731 |
| BMP Signaling | *Runx2* | 1426903_at | — | 4,6307 | 4,2644 |
| BMP Signaling | *Smurf1* | 1417658_at | — | 0,1330 | 0,7085 |
| BMP Signaling | *Msx1* | 1421907_at | — | 3,5765 | 3,6782 |
| BMP Signaling | *Msx2* | 1421909_at | — | 2,6547 | 4,4635 |
| BMP Signaling | *Dlx5* | 1427227_at | — | -0,2990 | 1,0477 |
| BMP Signaling | *Scx* | 1449774_at | — | 1,4385 | -0,8271 |
| BMP Signaling | *Megf8* | 1426087_at | — | 1,6269 | 0,2380 |
| BMP Signaling | *Ror2* | 1416382_at | — | 4,9529 | 4,1660 |
| BMP Signaling | *Comp* | 1417345_at | — | 3,0774 | 3,4121 |
| BMP Signaling | *Slc33a1* | 1428096_at | — | 4,5652 | 2,5491 |
| BMP Signaling | *Slc39a5* | 1422799_at | — | 5,3575 | 5,6753 |
| BMP Signaling | *Pdcd4* | 1417888_at | — | 1,9847 | 2,4542 |
| BMP Signaling | *Pou3f1* | 1421494_at | — | -1,3792 | 2,6014 |
| BMP Signaling | *Usp9x* | 1437287_at | — | 2,0501 | 2,4397 |
| BMP Signaling | *Usp15* | 1419074_at | — | 3,1071 | 2,4441 |
| BMP Signaling | *Hfe* | 1418015_at | — | -0,0102 | 2,5428 |
| BMP Signaling | *Mapk3* | 1417456_at | — | 3,9456 | 2,3440 |
| BMP Signaling | *Ddx5* | 1416488_at | — | 2,3015 | 1,6526 |
| BMP Signaling | *Zcchc15* | 1430518_at | — | 2,3875 | 0,6405 |
| BMP Signaling | *Etv2* | 1456101_at | — | 1,9578 | 0,5994 |
| BMP Signaling | *Wnt3a* | 1416003_at | L | 1,2369 | 2,1146 |
| **SHH Signaling** | | | | | |
| SHH Signaling | *Gli2* | 1435269_at | — | 2,5266 | 4,8692 |
| SHH Signaling | *Prkar1b* | 1416271_at | — | 5,8885 | 4,3006 |
| SHH Signaling | *Smo* | 1416529_at | R | 2,8615 | -0,1248 |
| SHH Signaling | *Ptch1* | 1449025_at | R | 5,8270 | 3,3806 |
| SHH Signaling | *Prkar2a* | 1416272_at | — | 6,0402 | 2,2376 |
| SHH Signaling | *Gli3* | 1426562_a_at | — | 1,8886 | 2,4293 |
| SHH Signaling | *Shh* | 1451553_at | L | 3,0954 | 4,1042 |
| SHH Signaling | *Prkacb* | 1415826_at | — | 3,3820 | 1,9308 |
| SHH Signaling | *Gli1* | 1417182_at | — | 3,8101 | 2,7560 |
| SHH Signaling | *Prkar1a* | 1416270_at | — | 5,9584 | 4,9617 |
| SHH Signaling | *Sufu* | 1418342_at | — | 4,8504 | 4,5140 |
| SHH Signaling | *Prkar2b* | 1416273_at | — | 4,0419 | 4,4161 |
| SHH Signaling | *Gsk3b* | 1460700_at | — | 3,5154 | 2,7749 |
| SHH Signaling | *Dyrk1b* | 1423588_at | — | 3,3924 | 2,3549 |
| SHH Signaling | *Dyrk1a* | 1423584_at | — | 7,2802 | 7,0299 |
